# Improved air-stability and conductivity in the 75Li_2_S·25P_2_S_5_ solid-state electrolyte system: the role of Li_7_P_3_S_11_†

**DOI:** 10.1039/d3ra04706g

**Published:** 2023-09-08

**Authors:** Chen Mi, Simon R. Hall

**Affiliations:** a School of Chemistry, University of Bristol Cantock's Close Bristol BS8 1TS UK simon.hall@bristol.ac.uk

## Abstract

Doping modification is regarded as a simple and effective method for increasing the ionic conductivity and air stability of solid state electrolytes. In this work, a series of (100−*x*)(0.75Li_2_S·0.25P_2_S_5_)·*x*P_2_O_5_ (mol%) (*x* = 0, 1, 2, 3 and 4) glass-ceramic electrolytes were synthesized by a two-step ball milling technique. Various characterization techniques (including powder X-ray diffraction, Raman and solid-state nuclear magnetic resonance) have proved that the addition of P_2_O_5_ can stimulate 75Li_2_S·25P_2_S_5_ system to generate the high ionic conductivity phase Li_7_P_3_S_11_. Through the doping optimization strategy, 98(0.75Li_2_S·0.25P_2_S_5_)·2P_2_O_5_ glass-ceramic (2PO) not only had a 3.6 times higher ionic conductivity than the undoped sample but also had higher air stability. Its ionic conductivity remained in the same order of magnitude after 10 minutes in the air. We further investigated the reasons why 2PO has a relatively high air stability using powder X-ray diffraction and scanning electron microscopy in terms of crystal structure degradation and morphology changes. In comparison to the undoped sample, the high ionic conductivity phases (β-Li_3_PS_4_ and Li_7_P_3_S_11_) of 2PO were better preserved, and less impurity and unknown peaks were generated over a short period of exposure time. In addition, the morphology of 2PO only changed slightly after 10 minutes of exposure. Despite the fact that the particles aggregated significantly after several days of exposure, 2PO tended to form a protective layer composed of S_8_, which might allow some particles to be shielded from attack by moisture, slowing down the decay of material properties.

## Abstract 2

We have investigated irregular material degradation using two different exposure methods. 98(0.75Li_2_S·0.25P_2_S_5_)·2P_2_O_5_ tended to form a protective layer, which might allow some particles to be shielded from attack by moisture, slowing down material decay.

## Graphical abstract

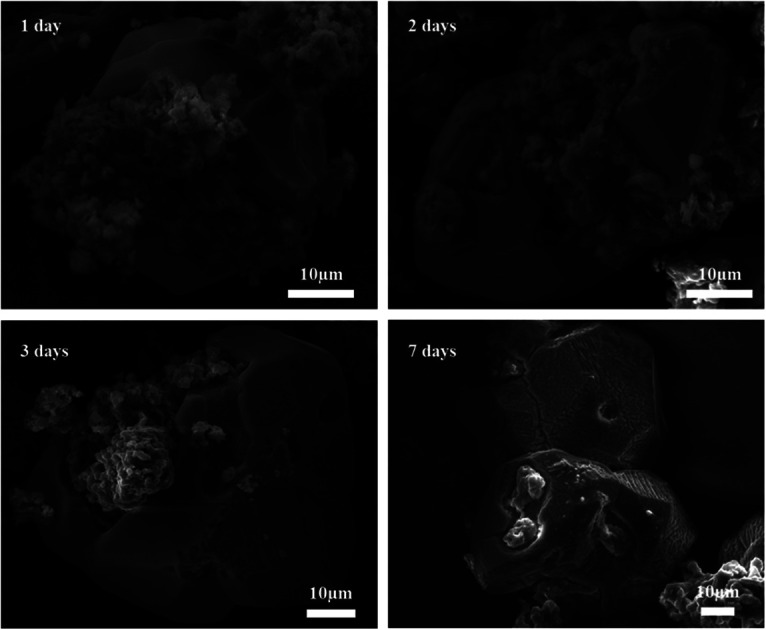

## Introduction

The rapid development of electric vehicles and power station energy storage devices has highlighted the need for developments in the safety of lithium-ion batteries (LIBs).^1^ At the moment, replacing traditional LIBs containing flammable liquid electrolytes with all-solid-state lithium-ion batteries (ASSLIBs) containing solid state electrolytes (SSEs) is regarded as one of the most effective and promising ways to enhance safety.^2^ Numerous investigations have shown that high-performance SSEs are essential for the outstanding performance of ASSLIBs.^3–5^ Therefore, the key component SSEs have attracted a lot of research interest as a foundation for developing new ASSLIBs.^6^

SSEs are typically classified into three classes based on their composition: inorganic solid electrolytes (ISEs), polymer solid electrolytes (PSEs) and composite solid electrolytes.^7^ Sulfide solid electrolyte is one of the most widely used ISEs owing to its high ionic conductivity, wide electrochemical stability window and high deformability.^8,9^ Li_2_S·P_2_S_5_ (LPS) system is one of the most representative materials in sulfide solid electrolytes. It is reported that 70Li_2_S·30P_2_S_5_ (mol%) glass-ceramic can reach the high ionic conductivity of 3.2 × 10^−3^ S cm^−1^ at room temperature (RT).^10^ However, when compared to the conductivity of traditional liquid electrolytes (up to 10^−2^ S cm^−1^),^2^ this is far from sufficient. Besides, the hygroscopicity of sulfide solid electrolytes severely limits their application. When exposed to air, their structure collapses and morphology deforms, and H_2_S gas is generated.^11,12^ As a result, researchers are attempting to improve their ionic conductivity and air stability using a variety of techniques. Doping is widely used for the modification of materials due to its advantages of simplicity and efficacy.^13–15^ For example, heteroatom-doped graphenes have a widely tunable work function,^16^ the catalytic activity of Mo-doped iron phosphide is significantly improved,^17^ and the doped anode^18^ and cathode materials^19,20^ can improve battery capacity effectively.

Doping oxygen into LPS in the form of various compounds is a common practice.^21–23^ Huang *et al.*^24^ synthesized 70Li_2_S·29P_2_S_5_·1Li_3_PO_4_ glass-ceramic with high ionic conductivity of 1.87 × 10^−3^ S cm^−1^ (RT), as well as an activation energy of 18 kJ mol^−1^. Tao *et al.*^25^ emphasized that the formation of a bridging oxygen atom after oxygen doping substitutes a non-bridging sulfur atom, lowering the activation energy needed for lithium ions to migrate and increasing the ionic conductivity of the material. By adding P_2_O_5_ to 70Li_2_S·30P_2_S_5_ glass-ceramic, a sulfide solid electrolyte with a high ionic conductivity of 2.61 × 10^−3^ S cm^−1^ was obtained.^26^ Studies have demonstrated that the addition of oxygen can improve not only ionic conductivity but also air stability.^11,27,28^ Ohtomo *et al.*^29^ proved that partially substituting Li_2_O for 75Li_2_S·25P_2_S_5_ glass can effectively limit H_2_S generation. Hayashi *et al.*^30^ reported that the composite electrolyte prepared by combining Li_3_PS_4_ (75Li_2_S·25P_2_S_5_) glass with metal oxides M_*x*_O_*y*_ (M_*x*_O_*y*_: Fe_2_O_3_, ZnO and Bi_2_O_3_) can successfully suppress the formation of H_2_S gas, enhancing the chemical stability of the electrolyte in humid air. Unfortunately however, most studies on the air stability of sulfide solid electrolytes are conducted by simply placing the material in an air-filled desiccator. Although this test very important for the preliminary evaluation of the air stability of the material, the experimental results are highly influenced by the operation of the researchers and provide little by way of understanding how the material changes chemically and morphologically in the air. With the continued focus on the air stability of sulfide solid electrolytes, an increasing number of researchers have conducted further in-depth research. Li *et al.*^31^ studied the structural changes of Li_3_PS_4_ and its modified material in the air for up to 2 hours using *in situ* X-ray diffraction (XRD). In addition, the Qing Jiao research group^32,33^ proposed an impactful explanation for the slow degradation of modified Li_3_PS_4_ using a variety of technologies including XRD, Raman, EIS (electrochemical impedance spectroscopy) and SEM (scanning electron microscopy). There remains however, little research on the enhancement of sulfide solid electrolytes air stability by way of doping. It is apparent that sulfide solid electrolytes with high air stability could not only simplify the assembly conditions of ASSLIBs but also improve their safety (considering the problem of H_2_S release). Therefore, in addition to having high ionic conductivity, it is necessary to develop sulfide solid electrolytes with high air stability, which also serves as the foundation for subsequent research on ASSLIBs. In addition, comprehending the degradation process of sulfide solid electrolytes will aid in the investigation of potential techniques to delay their decomposition.

In this work, we study the effect on the effect of P_2_O_5_ doping on the sulfide solid electrolyte 75Li_2_S·25P_2_S_5_ system. First, we successfully prepared (100−*x*)(0.75Li_2_S·0.25P_2_S_5_)·*x*P_2_O_5_ (mol%) (*x* = 0, 1, 2, 3 and 4) glass-ceramic based on the optimal synthesis conditions.^12^ We show how a small amount of P_2_O_5_ doping changes the structure of 75Li_2_S·25P_2_S_5_ system by using powder X-ray diffraction (pXRD), Raman and ^31^P magic angle spinning nuclear magnetic resonance spectroscopy (^31^P MAS NMR). Furthermore, we show that 98(0.75Li_2_S·0.25P_2_S_5_)·2P_2_O_5_ glass-ceramic has better air stability in comparison to 75Li_2_S·25P_2_S_5_ glass-ceramic without the addition of P_2_O_5_, in addition to having higher ionic conductivity. We further explore the changes in the ionic conductivity, crystal structure phases and morphology of 98(0.75Li_2_S·0.25P_2_S_5_)·2P_2_O_5_ glass-ceramic after exposure to air for a period of time through EIS, pXRD and SEM. Finally, we propose that substitution of 2 mol% P_2_O_5_ can slow the rate of ionic conductivity decline in 75Li_2_S·25P_2_S_5_ system in the air, and explain it from the perspective of crystal structure phases and morphology.

## Results and discussion

### Doping P_2_O_5_ changing the main structure of the 75Li_2_S·25P_2_S_5_ system

**Fig. 1:**
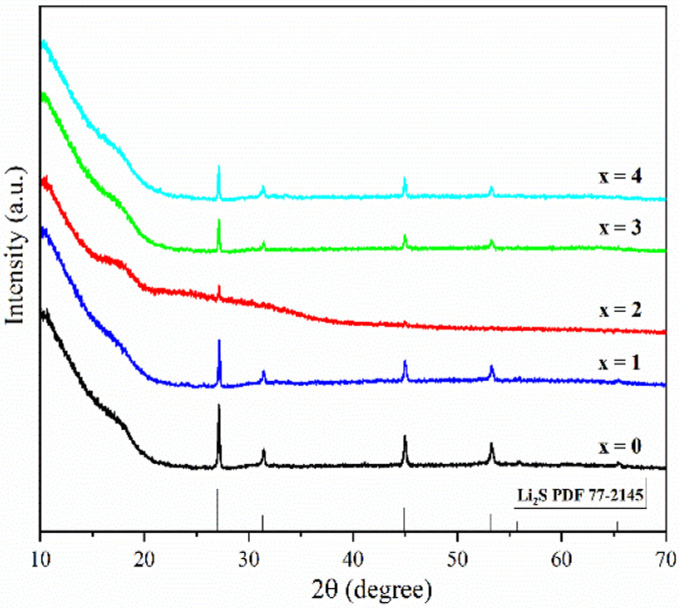
pXRD patterns of ball-milled (100−*x*)(0.75Li_2_S·0.25P_2_S_5_)·*x*P_2_O_5_ (*x* = 0, 1, 2, 3 and 4).

Fig. 1 shows the pXRD patterns of ball-milled (100−*x*)(0.75Li_2_S·0.25P_2_S_5_)·*x*P_2_O_5_ (*x* = 0, 1, 2, 3 and 4). Silicon bubble holders are used to protect the sample from moisture damage, which would result in a high background in the low angle range. The pXRD patterns of P_2_O_5_-doped ball-milled samples retained the Li_2_S crystal peaks, which was similar to the pattern of undoped sample.^12^ Although this unusual phenomenon has also been reported by some researchers,^27,29,34–37^ no explanation other than that related to the synthesis conditions has been proposed so far. It is worth noting that when *x* = 2, the sample has a relatively weak Li_2_S crystal peak, implying that doping with 2% P_2_O_5_ plays a role in the reduction of residual Li_2_S in the ball-milled 75Li_2_S·25P_2_S_5_ system. The pXRD patterns of all samples did not reveal any ZrO_2_ diffraction peaks or other impurity peaks, indicating that the sample was not contaminated by ball milling jars and balls or other unexpected contaminants.

**Fig. 2:**
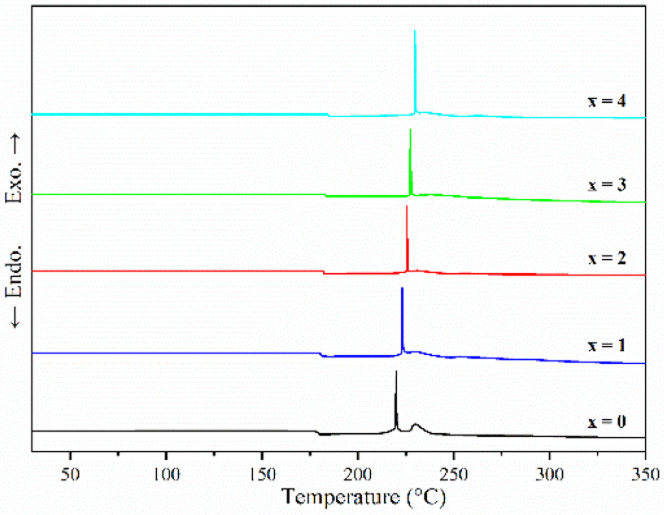
DSC curves of ball-milled (100−*x*)(0.75Li_2_S·0.25P_2_S_5_)·*x*P_2_O_5_ (*x* = 0, 1, 2, 3 and 4).

**Fig. 3:**
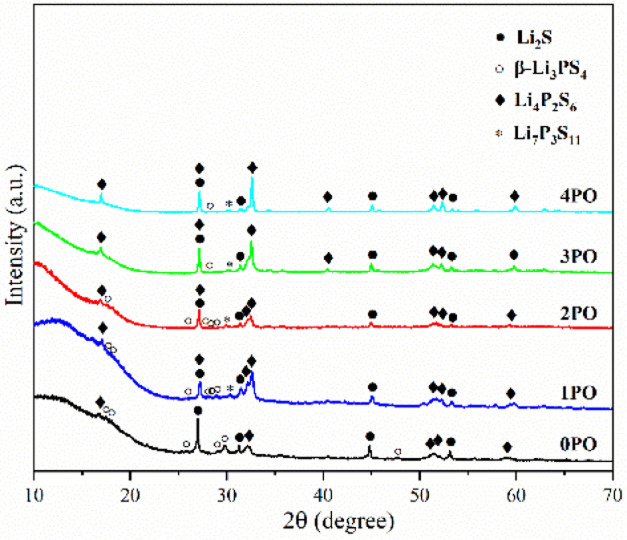
pXRD patterns of the 0PO, 1PO, 2PO, 3PO and 4PO.

**Fig. 4:**
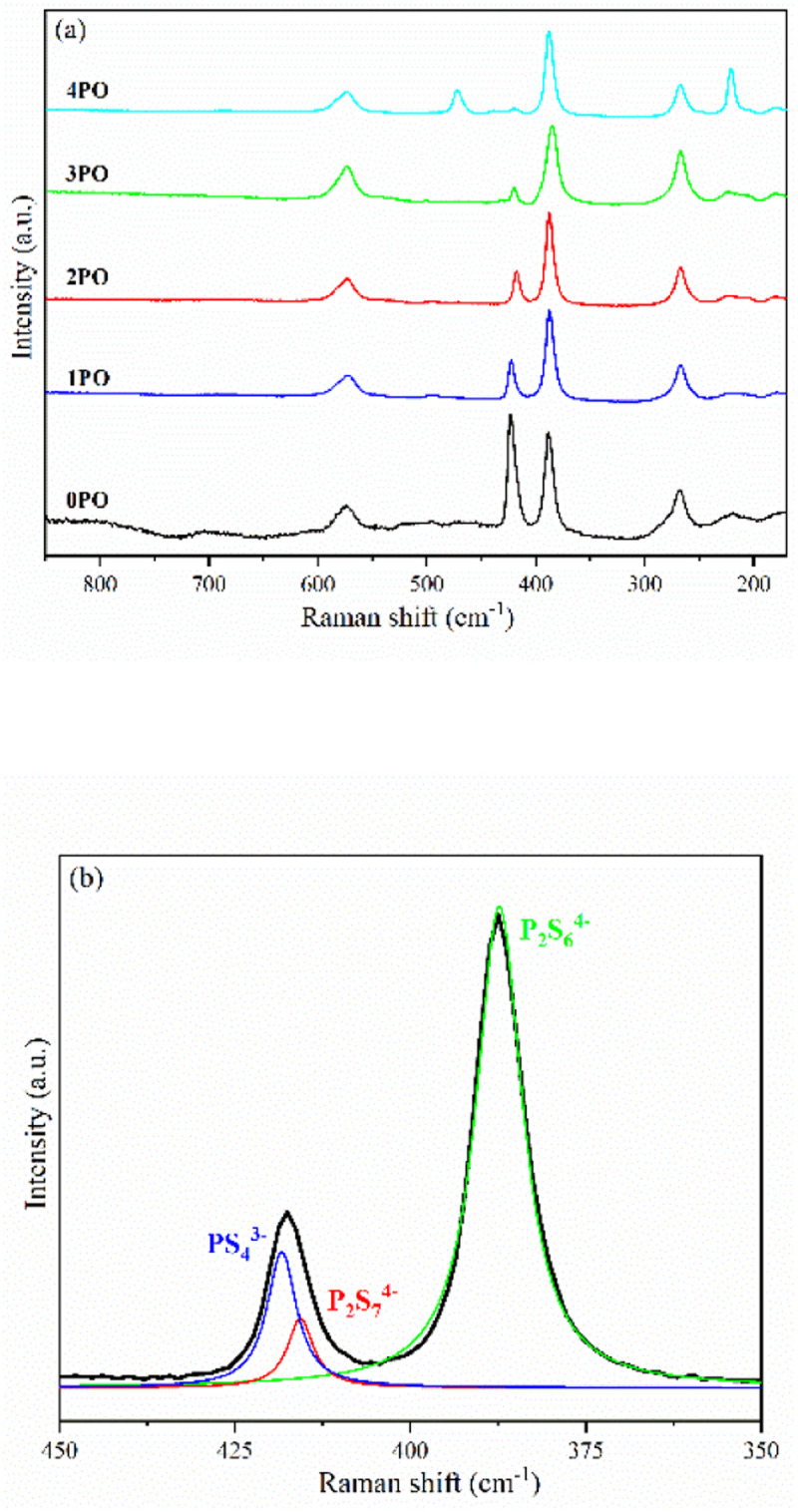
(a) Raman spectra of 0PO, 1PO, 2PO, 3PO and 4PO. (b) Deconvoluted Raman spectra of 2PO. The black line represents the experimental data. The blue, green and red lines are the deconvoluted signals attributed to PS_4_^3−^, P_2_S_6_^4−^ and P_2_S_7_^4−^ moieties, respectively.

**Fig. 5:**
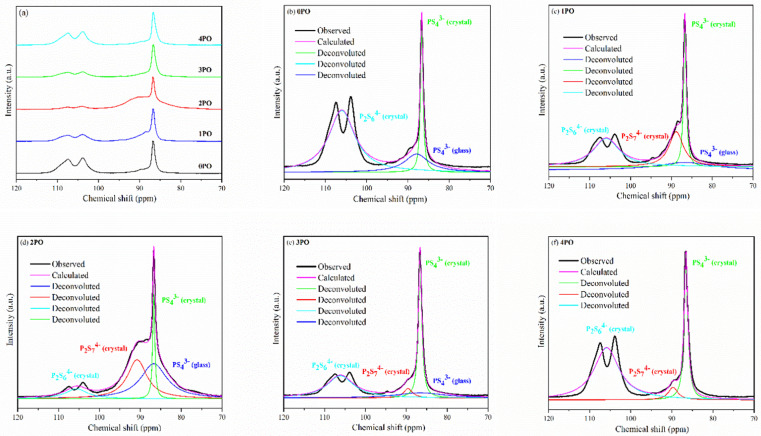
(a) ^31^P MAS NMR spectra of 0PO, 1PO, 2PO, 3PO and 4PO. The peak-deconvolution results for (b) 0PO, (c) 1PO, (d) 2PO, (e) 3PO and (f) 4PO. The fitting parameters of 0PO, 1PO, 2PO, 3PO and 4PO are shown in Tables S9–S13,† respectively.

Fig. 2 shows the DSC curves of ball-milled (100−*x*)(0.75Li_2_S·0.25P_2_S_5_)·*x*P_2_O_5_ (*x* = 0, 1, 2, 3 and 4) in the heating process. In all ball-milled samples, there is an endothermic change attributable to a glass transition, as well as one sharp exothermic peak associated with crystallization. These characteristic phenomena on the DSC curves indicate that all the ball-milled samples have glass-like properties.^38^ In addition, the glass transition temperature (*T*_g_) and first crystallization temperature (*T*_c_) shifted to the higher temperature side as the P_2_O_5_ percentage increased. As a general rule, one of the indications to determine the glass stability against crystallization is the difference between *T*_g_ and *T*_c_, that is *T*_c_ − *T*_g_.^39^ Table S1† presents the *T*_g_, *T*_c_ and *T*_c_ − *T*_g_ of all samples. The value of *T*_c_ − *T*_g_ increases from 41.7 °C to 45.2 °C as the proportion of P_2_O_5_ grows from 0 to 4 mol%, implying that a tiny quantity of P_2_O_5_ doping can improve the glass stability against crystallization of 75Li_2_S·25P_2_S, which is consistent with a previous report.^39^ Furthermore, when combined with the results of pXRD, Raman, and ^31^P MAS NMR (Fig. 3–5, discussed later), this is thermodynamic evidence for the formation of Li_7_P_3_S_11_ in the system, as the formation temperature of Li_7_P_3_S_11_ is higher than that of Li_3_PS_4_.^38^

For ease of notation, we will refer to annealed (100−*x*)(0.75Li_2_S·0.25P_2_S_5_)·*x*P_2_O_5_ (*x* = 0, 1, 2, 3 and 4) as 0PO, 1PO, 2PO, 3PO and 4PO, respectively. The pXRD patterns of 0PO, 1PO, 2PO, 3PO and 4PO can be seen in Fig. 3. For the first time, we discovered that adding P_2_O_5_ to the 75Li_2_S·25P_2_S_5_ system caused the formation of the Li_7_P_3_S_11_ phase with high ionic conductivity.^40^ One of the reasons for the formation of the Li_7_P_3_S_11_ phase is the addition of a small amount of P_2_O_5_ changes the proportions of Li, P, and S in the system. More specifically, the ratio of P to Li and S in Li_7_P_3_S_11_ (P/Li = 0.43, P/S = 0.27) is between Li_3_PS_4_ (P/Li = 0.33, P/S = 0.25) and Li_4_P_2_S_6_ (P/Li = 0.5, P/S = 0.33). As a result, an appropriate amount of P_2_O_5_ doping could generate a Li_7_P_3_S_11_ phase in the 75Li_2_S·25P_2_S_5_ system theoretically that originally contained a certain amount of impurities (Li_4_P_2_S_6_ and Li_2_S). The rapid loss of some Li and S with nitrogen flow during the heating process is largely responsible for the formation of low ion conductivity phase Li_4_P_2_S_6_,^41^ whereas the addition of P_2_O_5_ increases the proportion of P in the system to a certain extent, allowing Li and S to combine with P more quickly to reduce the loss, thereby changing the proportion of generated Li_*x*_P_*y*_S_*z*_ (Li_3_PS_4_, Li_7_P_3_S_11_ and Li_4_P_2_S_6_) phases in the system.

Fig. 4(a) shows the Raman spectra of 0PO, 1PO, 2PO, 3PO and 4PO. The peak assignments of these samples are list in Tables S2–S6,† respectively. With the addition of P_2_O_5_, the PS_4_^3−^ peak at 423 cm^−1^ shows a tendency to shift towards lower wavenumbers, which is attributed to the formation of the Li_7_P_3_S_11_ phase in the system. One of the Raman characteristic peaks of Li_7_P_3_S_11_ crystal is the P_2_S_7_^4−^ unit at 415 cm^−1^.^35^ Unfortunately, none of the doped samples have clearly separated PS_4_^3−^ and P_2_S_7_^4−^ peaks, which could be due to overlapping caused by their close proximity. According to Dietrich *et al.*,^38^ more details about the P_*x*_S_*y*_^*a*−^ (for example, P_2_S_6_^4−^, P_2_S_7_^4−^, PS_4_^3−^) units in the materials can be learned by deconvoluting the Raman range between 450 cm^−1^ and 350 cm^−1^ in these spectra. The deconvoluted Raman spectra of 1PO and 2PO are shown in Fig. S1† and 4(b), respectively. The corresponding fitting parameters are tabulated in Tables S7 and S8.† Since PS_4_^3−^ and P_2_S_7_^4−^ units both contribute to the peak around 420 cm^−1^, we do not specify the exact attribution of this peak in Tables S3–S6.† The intensity of the peak around 420 cm^−1^ is low, which is one reason we did not deconvolve the 3PO and 4PO in this region, however their conformity with the above conclusion can be confirmed (this is discussed below). The existence of S_8_ peaks can be identified in Raman spectra when the (100−*x*)(0.75Li_2_S·0.25P_2_S_5_)·*x*P_2_O_5_ glass-ceramic contains a high proportion of the impurity phase Li_4_P_2_S_6_ (such as 4PO), which conforms to the results of Ohtomo *et al.*^29^ and Hood *et al.*^42^

Solid-state NMR (ss-NMR) were used to further investigate the components and phases of prepared (100−*x*)(0.75Li_2_S·0.25P_2_S_5_)·*x*P_2_O_5_ glass-ceramic electrolytes. The ^31^P MAS NMR spectra of a series of doped glass-ceramic electrolytes are shown in Fig. 5(a), compared with the undoped sample. It can be seen that the doped samples exhibit nearly identical characteristic peaks, with a main peak of 86 ppm, a broad shoulder peak of 90 ppm, and split into two small peaks at 106 ppm, all of which are in good agreement with structural units PS_4_^3−^ + POS_3_^3−^ (where one sulfur of the PS_4_^3−^ structural motif is replaced with an oxygen atom correspondingly^25,29,39^), P_2_S_7_^4−^ and P_2_S_6_^4−^.^43–46^ The PS_4_^3−^ structural unit peak could eclipse the non-bridging POS_3_^3−^ unit peak in NMR spectra of doped electrolytes at around 86 ppm.^47^ It was proposed that the new oxysulfide unit (P_2_OS_6_^4−^) could be partially formed in the material after oxide doping, with the bridging sulfur (P–S–P) in the P_2_S_7_^4−^ unit being replaced by the doped bridging oxygen (P–O–P).^38,47^ The two split peaks at 106 ppm are the ss-NMR characteristic peaks of Li_4_P_2_S_6_ crystal, indicating that its crystal structure contains at least two distinct crystallographic phosphorus sites, in accordance with research done by Neuberger *et al.*^46^

A full structural analysis of glass-ceramic electrolytes reveals that they contain amorphous and crystalline phases to varying degrees.^48^ ss-NMR has been used as a powerful and cutting-edge technology to identify amorphous and crystalline phases in glass-ceramic electrolyte materials.^44^ According to our previous research on 75Li_2_S·25P_2_S_5_ glass-ceramic electrolyte,^12^ the main structural unit of the amorphous portion is PS_4_^3−^ with a small amount of P_2_S_6_^4−^, while the structural units PS_4_^3−^ and P_2_S_6_^4−^ exist in β-Li_3_PS_4_ and Li_4_P_2_S_6_ crystal, respectively. Some studies using various technologies prove that the highly conductive crystalline phase Li_7_P_3_S_11_ contains structural units of PS_4_^3−^ and P_2_S_7_^4−^.^10,40,44^ According to the above pXRD and Raman results, the structural units of PS_4_^3−^ (glass), PS_4_^3−^ (crystal), P_2_S_6_^4−^ (glass + crystal) and P_2_S_7_^4−^ (crystal) should be considered in the doped electrolytes. Fig. 5(b)–(f) show the ^31^P MAS NMR spectra of the (100−*x*)(0.75Li_2_S·0.25P_2_S_5_)·*x*P_2_O_5_ (*x* = 0, 1, 2, 3 and 4) glass-ceramic electrolytes after deconvolution separately.^44,49^ The corresponding fitting parameters are tabulated in Tables S9–S13,† respectively. The absence of PS_4_^3−^ (glass) in 4PO could be attributed to the high proportion of Li_4_P_2_S_6_ crystal impurity in the material.^29,42^ Additionally, the proportion of Li_7_P_3_S_11_ crystal could be determined by calculating the degree of crystallization (*X*_c_)^50^

1 *X*_c_ (mol%) = *Φ*_P_2_S_7_^4−^_/*Φ*_all_ × 100

In this equation, *Φ*_P_2_S_7_^4−^_ represents the intensity (area) of P_2_S_7_^4−^ (crystal) peak, while *Φ*_all_ is the total intensity (area) of the resonance peaks. Similarly, the proportion of P_2_S_6_^4−^ (glass + crystal) (*Y*) in the system is defined as follows:

2 *Y* (mol%) = *Φ*_P_2_S_6_^4−^ (glass+crystal)_/*Φ*_all_ × 100

*Φ*_P_2_S_6_^4−^ (glass+crystal)_ represents the intensity (area) of P_2_S_6_^4−^ (glass + crystal) peak. The *X*_c_ and *Y* of all doped samples are shown in the Table 1, and 2PO possessed the highest *X*_c_ of 36.2%, the lowest *Y* of 10.1%. It is worth noting that, since we did not distinguish between P_2_S_6_^4−^ in amorphous and crystalline components, the *Y* obtained accounts for the combined contribution of the two components. Notable changes in structural unit intensity were observed in these ^31^P spectra with various amounts of P_2_O_5_ doping, indicating that the proportion of the high ionic conductivity Li_7_P_3_S_11_ phase in the 75Li_2_S·25P_2_S_5_ system is effectively optimized by the doping strategy.

**Table 1:** The degree of crystallization (*X*_c_) of 1PO, 2PO, 3PO and 4PO. *Φ*_P_2_S_7_^4−^_, *Φ*_P_2_S_6_^4−^ (glass+crystal)_ and *Φ*_all_ of all doped samples are obtained or calculated from Tables S10–S13

Sample	*Φ*_P_2_S_7_^4−^_	*Φ*_P_2_S_6_^4−^ (glass+crystal)_	*Φ*_all_	*X*_c_ (%)	*Y* (%)
1PO	1.6	2.4	6.5	24.6	36.9
2PO	2.5	0.7	6.9	36.2	10.1
3PO	0.3	1.9	4.6	6.5	41.3
4PO	0.4	4.2	6.3	6.3	66.7

### Ionic conductivity and air stability of the P_2_O_5_-doped 75Li_2_S·25P_2_S_5_ system

**Fig. 6:**
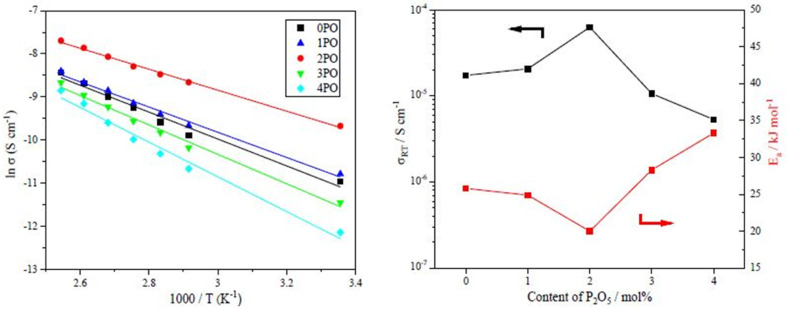
(a) The temperature dependence of ionic conductivities for the 0PO, 1PO, 2PO, 3PO and 4PO. (b) The ionic conductivities (RT) and activation energy of various P_2_O_5_ contents.

The performance of sulfide solid electrolyte materials is typically evaluated from two aspects: ionic conductivity and air stability.^11,26,36^ The temperature dependence of the ionic conductivity of the 0PO, 1PO, 2PO, 3PO, and 4PO is shown by the Arrhenius plot in Fig. 6(a). Fig. 6(b) shows the ionic conductivity (RT) and activation energy of various P_2_O_5_ contents. As expected, 2PO possess the highest ionic conductivity of 6.3 × 10^−5^ S cm^−1^ (RT) (about 3.7 times that of 0PO^12^) and the lowest activation energy of 20.0 kJ mol^−1^ calculated by the slope of the Arrhenius plot. It is reported that Li_7_P_3_S_11_ phase (about 10^−3^ S cm^−1^ at RT) has higher ionic conductivity than β-Li_3_PS_4_ phase (about 10^−4^ S cm^−1^ at RT),^35^ and the ionic conductivity of the Li_4_P_2_S_6_ crystal is around 10^−7^ S cm^−1^ at RT, which is a low ionic conductivity phase.^42^ According to the ss-NMR results, the main reason 2PO has the best ionic conductivity is that it contains the most of the high ionic conductivity phase Li_7_P_3_S_11_ and the least amount of the low ionic conductivity phase Li_4_P_2_S_6_. Although 1PO has a relatively high Li_7_P_3_S_11_ proportion, its ionic conductivity is almost unchanged when compared to undoped sample due to a relatively high Li_4_P_2_S_6_ proportion as well. In sharp contrast, 3PO and 4PO contain a significant amount of Li_4_P_2_S_6_ and only tiny amount of Li_7_P_3_S_11_ due to excessive addition of P_2_O_5_, causing their ionic conductivities to rapidly decrease. The Nyquist plot of electrochemical impedance for the 2PO at various temperatures is shown in Fig. S2.† The value of *Z*′ at the intercept with the real axis determined by linear fitting was used to estimate the resistance.^35,51^ The results are presented in Table S14.†

**Fig. 7:**
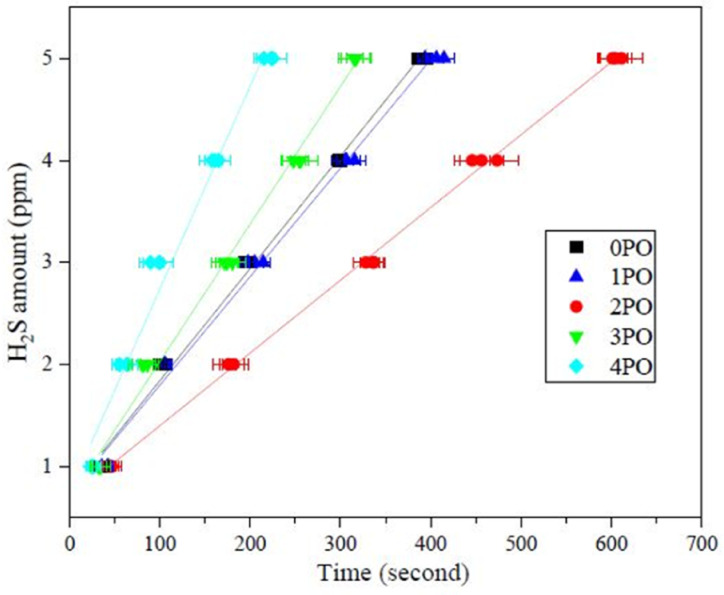
Amounts of H_2_S released from 0PO, 1PO, 2PO, 3PO and 4PO after exposure to air.

Another crucial aspect of evaluating sulfide solid electrolyte performance is its stability in the air. It is important to note that, due to the inherent properties of sulfide solid electrolytes,^11,28^ the air stability of the doped samples discussed in this paper is relative to the undoped sample. The amounts of H_2_S released from doped samples after exposure to air in comparison to an undoped sample are shown in Fig. 7. It is clear that 2PO has a higher level of air stability, taking longer to release the same amount of H_2_S. The release of 5 ppm H_2_S (alarm value of gas sensor) takes approximately 10 min (∼615 seconds) (air temperature: 20–25 °C, relative humidity: around 70%).

**Fig. 8:**
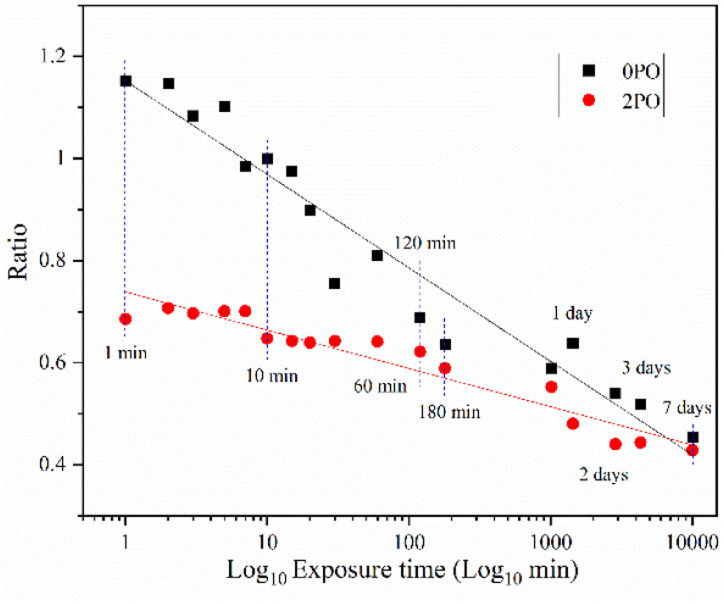
The evolution of the S element percentage of 0PO and 2PO over time. The percentage change is normalized to the carbon peak.

EDS was used to further investigated the stability of 2PO from the perspective of changes in element content. The EDS spectra of 2PO exposed at various times in Fig. S3† reflects that, with the exception of elements lithium and hydrogen, which are not detectable by EDS, the obtained degradation electrolytes contain elements sulfur, phosphorus and oxygen (the element carbon from graphite coating). Fig. 8 shows the evolution of the S element percentage identified by EDS over time, with 0PO serving as a comparison. As expected, the content of S in both materials showed a downward trend with the release of H_2_S. It is worth noting that the decreased rate of S element percentage in 2PO is significantly slower than that of 0PO, further demonstrating that the P_2_O_5_-doped 75Li_2_S·25P_2_S_5_ system does have higher stability in the air than the undoped system.

### Degradation of 98(0.75Li_2_S·0.25P_2_S_5_)·2P_2_O_5_ glass-ceramic (2PO)

**Fig. 9:**
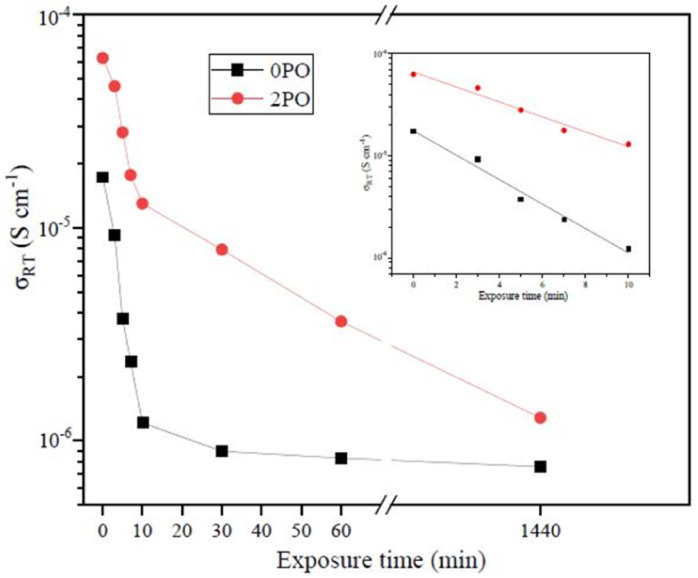
The ionic conductivity (RT) of 0PO and 2PO electrolytes at various exposure times.

Fig. 9 shows the ionic conductivity (RT) of 0PO and 2PO electrolytes at various exposure times. As expected, their conductivities decreased to varying degrees as exposure time increased. It is interesting to note that 2PO does have a slow decay trend in the first 10 min exposure time (the inset in Fig. 9), and that its ionic conductivity after 10 min of exposure (1.3 × 10^−5^ S cm^−1^) is still in the same order of magnitude as the unexposed sample. On the contrary, the conductivity of 0PO had significantly decreased. When exposed to air for more than 30 min, the ionic conductivity of 0PO appeared to change little because it has been as low as 10^−7^ S cm^−1^, which is at the lower range of ionic conductivity of SSEs.^42^ The EIS experimental results reveal that our doping and optimization strategy is effective in increasing the air stability of 75Li_2_S·25P_2_S_5_ system.

**Fig. 10:**
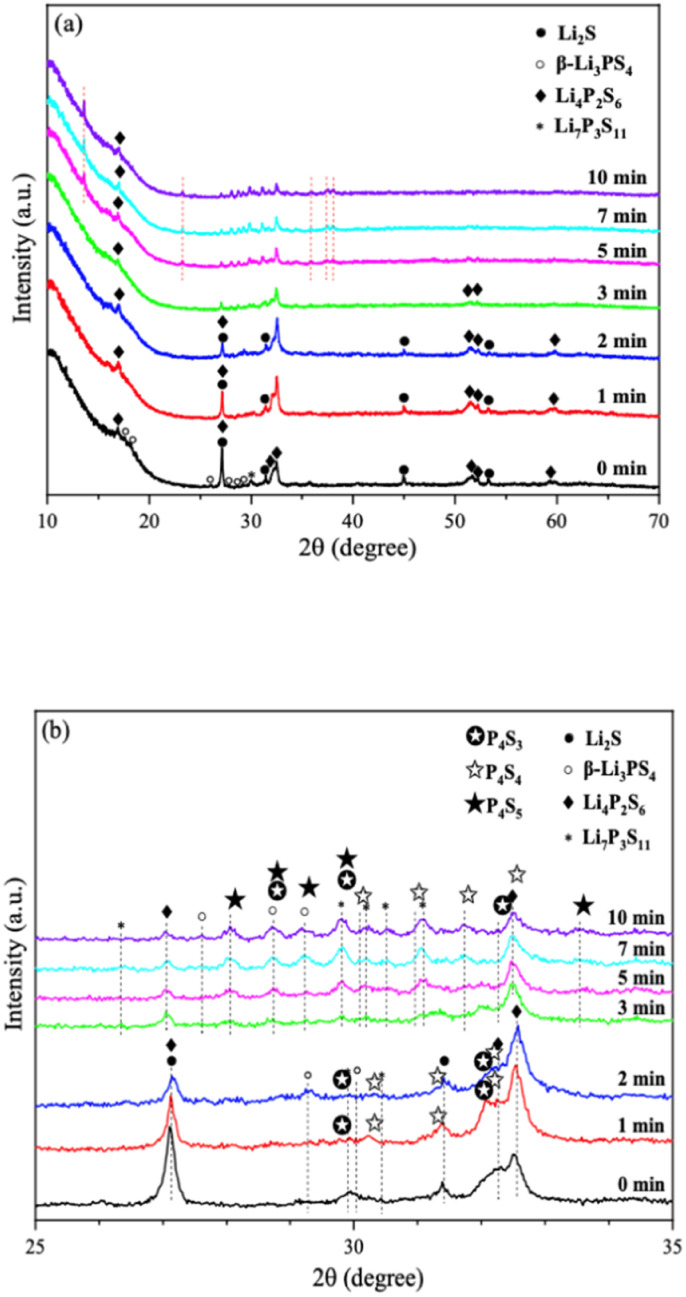
(a) pXRD patterns of 2PO exposed to air for 1 min, 2 min, 3 min, 5 min, 7 min and 10 min (b) is an enlarged view of (a) in the 2*θ* of 25° to 35° range.

There has always been a significant gap in research on the degradation of sulfide solid electrolytes. The irregular and complex degradation process of electrolytes increases the difficulty of tracking and characterization, as well as safety concerns for researchers. In order to fill a gap in the literature, this work employs novel exposure methods to capture the degradation status of materials under relatively short exposure times and long-term exposure. We performed pXRD characterization on the above samples to investigate the possible reasons for the difference in the rate of ionic conductivity decline from the perspective of the phase composition of materials (see the ESI† for a detailed description of the sample preparation, named exposure method A for pXRD). Fig. 10(a) shows the pXRD patterns of 2PO at various short exposure times. Fig. 10(b) is an enlarged view of Fig. 10(a) in the 2*θ* of 25° to 35° range. The pXRD patterns of 2PO did not change significantly after 2 min of exposure. The gradual weakening of the Li_2_S phase and the formation of tiny peaks attributed to the P_4_S_3_ and P_4_S_4_ phases, on the other hand, demonstrated that the internal structure of the material changed with the generation of H_2_S in a very short time after contact with moisture. The generation of P_4_S_3_ and P_4_S_4_ phases is most likely due to moisture causing the decomposition of the original phases β-Li_3_PS_4_, Li_7_P_3_S_11_ and/or Li_4_P_2_S_6_. After 3 minutes, the pXRD patterns become more complex, with more degradation phases and small associated peaks. Additionally, unknown diffraction peaks (red dashed line in Fig. 10(a)) appeared at roughly 13°, 22° and 38° from the 5 min exposure of the 2PO. These peaks could be the result of prolonged contact between the original phases and/or degradation phases and the H_2_O molecule in the atmosphere.^32^ Surprisingly, high ionic conductivity β-Li_3_PS_4_ and Li_7_P_3_S_11_ phases are still present in the material after 10 min, which could explain the slow conductivity decline of 2PO.

In addition, we also performed pXRD characterizations on the 0PO sample for comparison, as shown in Fig. S4.† In the first two minutes, the crystal phase change of 0PO was similar to that of 2PO. The Li_2_S phase gradually disappeared, giving rise to the formation of the P_4_S_3_, P_4_S_4_, and P_4_S_5_ phases. However, the unknown peak appeared at 34.5° from the first minute of exposure, and more unknown peaks appeared later at around 13°, 22°, 38° and 40°, indicating that the structure of undoped sample is easier to degrade and collapse than that of 2PO, which might additionally demonstrate that P_2_O_5_ can effectively alleviate the decomposition of the main structural units of materials. In addition, the high ionic conductivity phase β-Li_3_PS_4_ vanished after the second minute of exposure, which may also be strong evidence of its rapid decline in ionic conductivity. Therefore, the 75Li_2_S·25P_2_S_5_ system doped with P_2_O_5_ contributes to the relative stability of the material during air degradation due to the formation of the Li_7_P_3_S_11_ phase in the original main structure β-Li_3_PS_4_, which was previously not found in pure phase 75Li_2_S·25P_2_S_5_ doped system.^23,30,52,53^

**Fig. 11:**
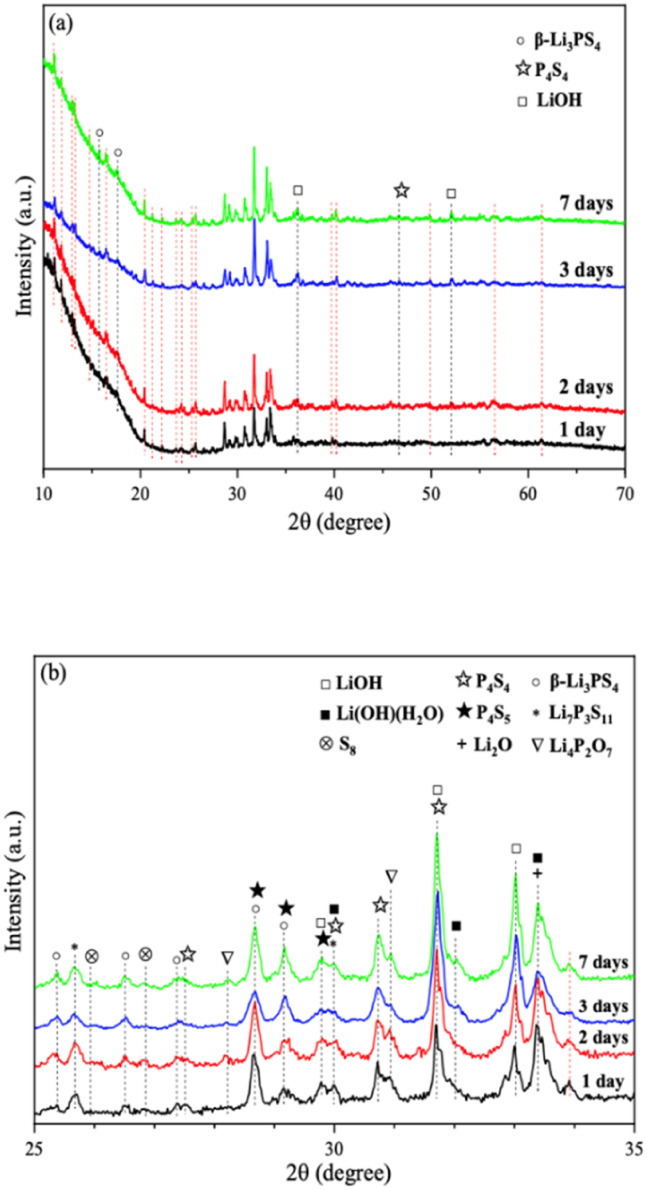
(a) pXRD patterns of 2PO exposed to air for 1 day, 2 days, 3 days, and 7 days (b) is an enlarged view of (a) in the 2*θ* of 25° to 35° range.

We further extended the exposure time of 2PO in the air. The pXRD patterns of 2PO exposed to the ambient environment for 1 day, 2 days, 3 days, and 7 days are shown in Fig. 11 (see the ESI† for a detailed description of the sample preparation, named exposure method B for pXRD). Fig. 11(b) is an enlarged view of Fig. 11(a) in the 2*θ* of 25° to 35° range. When compared to samples that had undergone short exposure, the pXRD patterns have almost completely changed, with more impurity phases such as S_8_, Li_2_O and LiOH and a plethora of unknown phases appearing, indicating that the material has almost completely degraded. This result shows some agreement with the findings of Li *et al.*^31^ and is essentially similar to the products produced by undoped sample exposed to air for an extended period of time.^12^ It is worth mentioning that Li *et al.*^31^ used a solution method to disperse Li_3_PS_4_ into the polymer (glycidyl methacrylate) matrix. Their synthesized sample possesses relatively strong air stability, with no significant change in XRD patterns after 20 min of exposure at 20% relative humidity. One of the important reasons for this seemingly large difference between that study and our work could be that our experimental environment is closer to the relative humidity in the ambient environment (∼70%).^54,55^ An advantage of our method over those previous studies is the simplicity of the doping method when compared with the solution method.^56^

The morphology changes of 2PO were investigated using two different exposure methods. The purpose of exposure method A is attempting to continually track how the morphology of an electrolyte exposed to a limited oxygen environment changes over a period of short time. In contrast, exposure method B is focussed on observing changes in the morphology of the electrolyte after prolonged exposure to the real ambient environment (see the ESI† for a detailed description of exposure methods A and B).

**Fig. 12:**
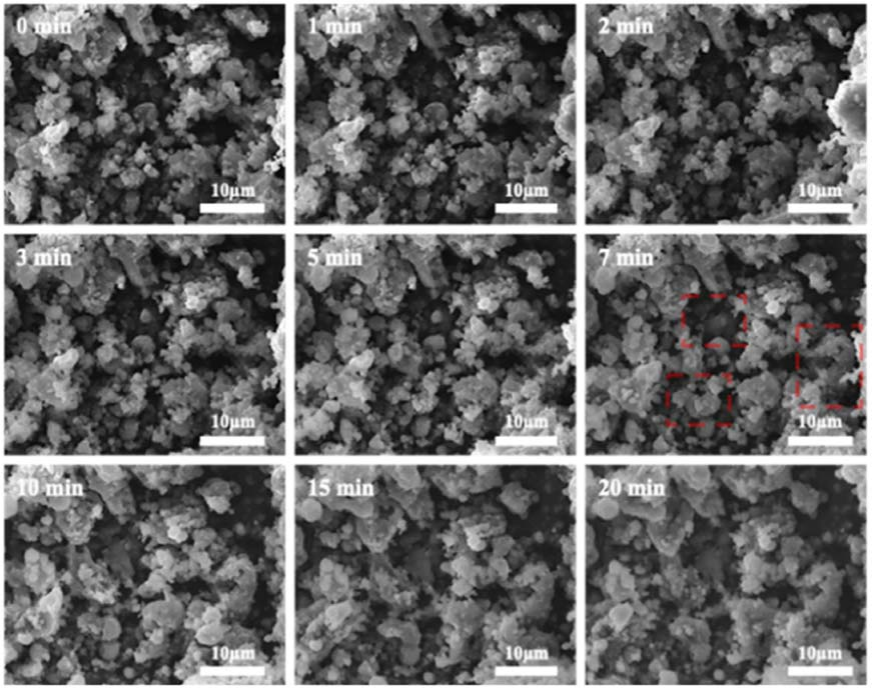
Typical SEM images of 2PO at various exposure times using exposure method A.

Fig. 12 shows SEM images of 2PO microstructure evolution at various exposure times using exposure method A. The 2PO electrolyte with no exposure (0 min) is primarily composed of irregular particles with particle sizes between 3 μm and 10 μm. EDS images of selected region (Fig. S5†) show that S, P and O elements are distributed uniformly throughout the electrolyte. The morphology of the electrolyte remained relatively stable for a period of time before changing slightly after 7–10 min of exposure. Some primary particles can be seen to aggregate to form larger secondary particles (especially in the region highlighted by the red square) with particle sizes between 8 μm and 15 μm. The aggregation progressed over time, and the sample had essentially lost its original main morphological characteristics after 20 min. This morphological change occurred because the electrolyte reacted with moisture in the air. It is possible that the larger irregular particles produced by aggregation could increase the transmission distance of lithium ions, which could also induce the ion transmission path to become tortuous, resulting in higher ionic conduction resistance, possibly serving as a further reason for the rapid decline in ionic conductivity of 2PO after 10 min.^57–59^ Fig. S6† shows additional evidence of a similar change trend in another test to further rule out the possibility that the change in sample morphology may be due to the influence of ‘charging’. We further increased the exposure time of 2PO in the air until it reached 17 h (1020 min). Videos of these experiments (Videos 1 and 2†) are included in the ESI,† corresponding to Fig. 12 and S6,† respectively.

**Fig. 13:**
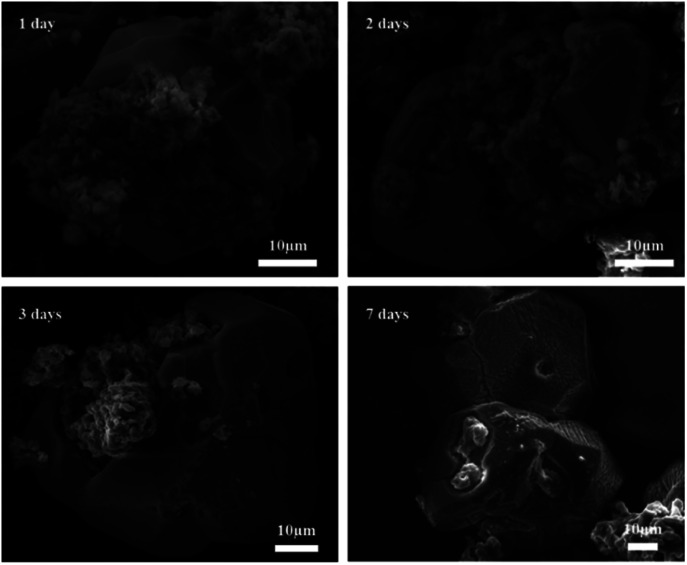
Typical SEM images of 2PO at various exposure times using exposure method B.

**Fig. 14:**
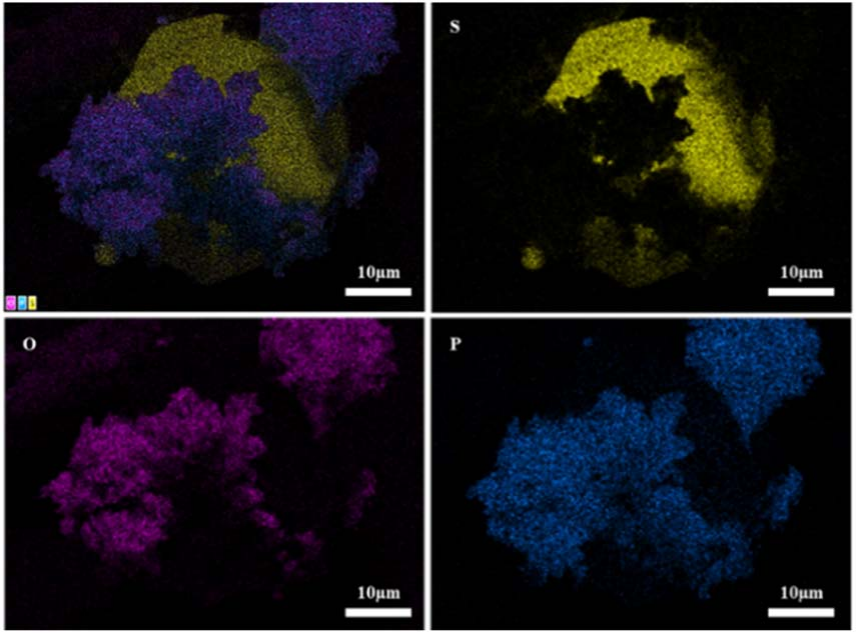
EDS elemental mapping of 2PO exposed to air for 1 day.

Fig. 13 shows SEM images of 2PO at various exposure times using exposure method B. We used different magnifications to show the entire morphology of the aggregated particles in the selected area. Some unusual morphological features can be noted. The sample that was exposed for 1 day displayed two distinct morphologies. On the one hand, some small irregular particles could still be detected despite the fact that particles have aggregated. On the other hand, a protective layer encapsulated these aggregated particles to form quasi-circular large particles with a diameter of around 40 μm. EDS was performed to further investigate the composition of this protective layer (as shown in Fig. 14). Obviously, this protective layer is mainly elemental sulfur (S_8_), which is consistent with the pXRD result (Fig. 11). Although the role of this feature in improving the air stability of solid electrolytes has not been thoroughly investigated, one explanation could be that the particles that are first attacked by the moisture tend to form an elemental sulfur protective layer to protect the particles that have not yet been attacked by the moisture or to slow the degradation rate of these particles. The presence of the similar protective layer was not detected in the undoped sample,^12^ which could explain the relatively high air stability and slow decrease in ionic conductivity of the 2% P_2_O_5_ doped sample. In addition, Wang *et al.*^32^ have proposed the concept of the protective layer as well and demonstrated that it may play a role in slowing down material degradation. The protective layer morphology can still be detected in the sample after 2 days of exposure, but the protective layer cracks, exposing more particles to air for degradation. The EDS of the sample exposed to air for 2 days is shown in Fig. S7.† As the exposure duration increases, the sample degrades entirely and more S_8_ is generated. Fig. S8† shows the SEM and EDS images of the sample after 7 days in the air. Furthermore, the surface of S_8_ in the samples exposed for 1 and 2 days was relatively smooth and soft, whereas the samples exposed for 3 and 7 days showed etching patterns, indicating that the samples had various degrees of degradation.

## Conclusions

We successfully synthesized a series of (100−*x*)(0.75Li_2_S·0.25P_2_S_5_)·*x*P_2_O_5_ (mol%) (*x* = 0, 1, 2, 3 and 4) glass-ceramic electrolytes by two-step ball milling technique. pXRD, Raman, and ss-NMR analyses revealed that adding P_2_O_5_ to 75Li_2_S·25P_2_S_5_ system with a main structure of β-Li_3_PS_4_ and a small amount of impurity Li_4_P_2_S_6_ can stimulate the formation of the high ionic conductivity phase Li_7_P_3_S_11_. Through the doping optimization strategy, 2% doped P_2_O_5_, that is 98(0.75Li_2_S·0.25P_2_S_5_)·2P_2_O_5_ glass-ceramic (2PO), had a 3.6 times higher ionic conductivity than the undoped sample due to a higher proportion of Li_7_P_3_S_11_. When the doping was excessive, however, the Li_4_P_2_S_6_ phase with low ionic conductivity dominated, severely reducing the ionic conductivity of the material. In addition to the increased ionic conductivity, 2PO also has relatively high air stability, which means that for the same amount of sample, 2PO releases less H_2_S than 0PO in the same amount of time. In addition, the ionic conductivity of 2PO remained in the same order of magnitude after 10 minutes in the air. pXRD and SEM were used to further investigated the reasons why 2PO has a relatively high air stability in terms of crystal structure degradation and morphology changes. In comparison to the undoped sample, the Li_7_P_3_S_11_ generated in 2PO contributes to maintain the stability of the main structure of the system over a short exposure time. Although some impurity phases form gradually, they are less than those generated in undoped samples. Doped and undoped samples produce similar final products after prolonged exposure. From the perspective of morphology, 2PO only changed slightly after a short exposure time, and aggregation caused by moisture attack is one of the reasons for the deterioration of material properties. The addition of P_2_O_5_ made 2PO tend to form an elemental sulfur protective layer after prolonged exposure, which might allow some particles to be shielded from the effects of moisture, slowing down the decay of material properties. This work closes a gap in research on the degradation of sulfide solid electrolytes and lays the foundation for improving the air stability of materials.

## Experimental

### Material synthesis

(100−*x*)(0.75Li_2_S·0.25P_2_S_5_)·*x*P_2_O_5_ (mol%) (*x* = 0, 1, 2, 3 and 4) glass-ceramic electrolytes were synthesized using a two-step ball milling technique (mechanical ball milling and subsequent heat treatment). The raw materials, including Li_2_S (Sigma Aldrich, 99.98%), P_2_S_5_ (Sigma Aldrich, 99%) and P_2_O_5_ (Sigma Aldrich, 99%), were weighed according to their respective molar ratios. Subsequently, the raw materials were transferred into a zirconia jar along with 15 zirconia balls (3 balls of 10 mm diameter and 12 balls of 5 mm diameter). The weight ratio of ball to powder was about 15 : 1. Then, the ball mill jar was placed in a planetary ball mill apparatus (CHANGSHA SAMY INSTRUMENT & EQUIPMENT CO. LTD, SQM-0.4L). The rotating speed was settled at 370 rpm for 48 hours. An agate mortar and pestle were used to further grind the ball-milled samples. Afterwards, the obtained ball-milled electrolyte powders were annealed at 260 °C in a tube furnace (Eurotherm, 2216E) for 3.5 h with a 5 °C min^−1^ heating rate. For ease of notation, we referred to these annealed samples as 0PO, 1PO, 2PO, 3PO and 4PO, respectively. All the preparation processes were performed in an N_2_ atmosphere.

### Material characterization

Powder X-ray diffraction was carried out on the samples using a Bruker D8 advance diffractometer with CuKα radiation to determine the crystal structural state of the electrolytes. The range of diffraction data was from 10° to 70° with 0.02° step size. Differential scanning calorimetry (DSC, TA DSC250) was performed to evaluate the thermal behaviour of samples. The test temperature ranged from 30 °C to 350 °C with a rate of 1 °C min^−1^. The Raman structure of electrolyte samples was measured using a Renishaw in via-reflex Raman spectrophotometer under the wavelength of 514.5 nm. The Raman shift varied between 850 cm^−1^ and 170 cm^−1^. Raman spectra were deconvoluted in the range between 450 cm^−1^ and 350 cm^−1^ by least squares data fitting based on Lorentz product functions using Origin Software of OriginLab. Magic angle spinning nuclear magnetic resonance (MAS NMR) spectroscopy were carried out on a Bruker Advance III spectrometer and a 4 mm probe operating at 162.01 MHz to study ^31^P. The samples were spun at approximately 13 kHz. The recycle delay was set to 50 seconds in order to obtain quantitative spectra of glass-ceramic samples. MAS NMR spectra were deconvoluted by least squares data fitting based on Lorentz product functions using Origin Software of OriginLab. The characteristic morphology of prepared materials was studied using a scanning electron microscope (SEM, JEOL IT300 SEM, Japan) assembly with energy dispersive spectroscopy (EDS). The samples were sputter coated with graphite prior to imaging.

In order to meet the requirements of safety and controllability, we used a 700 cm^3^ sealed desiccator filled with humid air to simulate the sample being placed in the air. The powder sample (0.05 g), a H_2_S gas sensor (GSR-04-EA-Z, Crowcon Gasman) and a fan were placed in the desiccator. The amount of H_2_S_(g)_ produced by electrolytes was measured constantly over a period of time. The air temperature was 20–25 °C and the relative humidity was around 70%.

### Electrochemical characterization

The impedance of cold pressed pelletized samples (pressed at 2 tons for 5 minutes) with 10 mm diameter and 1 mm thickness was measured using an alternating current (AC) impedance method. Stainless steel was employed as blocking electrodes on both sides of the pellet. The electrochemical impedance spectroscopy (EIS) was recorded using a CS2350 Bipotentiostat (Corrtest Instruments) between the frequency range from 1 MHz to 0.01 Hz with an excitation signal of 10 mV over a temperature range of 25–120 °C by using a Heratherm oven (Thermo Fisher Scientific). The calculation of ionic conductivity (*σ*) and activation energy (*E*_a_) of the samples is detailed in the ESI.†

## Author contributions

C. Mi synthesized and characterized all the samples mentioned in the manuscript. S. R. Hall supervised the project. C. Mi wrote the manuscript. All the authors discussed the results and reviewed the manuscript.

## Conflicts of interest

There are no conflicts to declare.

## Supplementary Material

RA-013-D3RA04706G-s001

## References

[cit1] Armand M., Tarascon J.-M. (2008). Nature.

[cit2] Du M., Liao K., Lu Q., Shao Z. (2019). Energy Environ. Sci..

[cit3] Fan W., Jiang M., Liu G., Weng W., Yang J., Yao X. (2022). ACS Appl. Mater. Interfaces.

[cit4] Zhang Q., Wan H., Liu G., Ding Z., Mwizerwa J. P., Yao X. (2019). Nano Energy.

[cit5] Zhang Q., Ding Z., Liu G., Wan H., Mwizerwa J. P., Wu J., Yao X. (2019). Energy Storage Mater..

[cit6] Tan G., Wu F., Zhan C., Wang J., Mu D., Lu J., Amine K. (2016). Nano Lett..

[cit7] Chen R., Qu W., Guo X., Li L., Wu F. (2016). Mater. Horiz..

[cit8] Xu X., Hou G., Nie X., Ai Q., Liu Y., Feng J., Zhang L., Si P., Guo S., Ci L. (2018). J. Power Sources.

[cit9] Nakamura H., Kawaguchi T., Masuyama T., Sakuda A., Saito T., Kuratani K., Ohsaki S., Watano S. (2020). J. Power Sources.

[cit10] Mizuno F., Hayashi A., Tadanaga K., Tatsumisago M. (2006). Solid State Ionics.

[cit11] Ohtomo T., Hayashi A., Tatsumisago M., Kawamoto K. (2013). J. Mater. Sci..

[cit12] Mi C., Hall S. R. (2023). Solid State Ionics.

[cit13] Hayashi A., Minami K., Tatsumisago M. (2010). J. Solid State Electrochem..

[cit14] Minami K., Hayashi A., Ujiie S., Tatsumisago M. (2011). Solid State Ionics.

[cit15] Rangasamy E., Liu Z., Gobet M., Pilar K., Sahu G., Zhou W., Wu H., Greenbaum S., Liang C. (2015). J. Am. Chem. Soc..

[cit16] Gholizadeh R., Yu Y.-X. (2014). J. Phys. Chem. C.

[cit17] Wu J., Li J.-H., Yu Y.-X. (2021). Catal. Sci. Technol..

[cit18] Yu Y.-X. (2013). Phys. Chem. Chem. Phys..

[cit19] Mi C., Han E., Li L., Zhu L., Cheng F., Dai X. (2018). Solid State Ionics.

[cit20] Mi C., Han E., Sun L., Zhu L. (2019). Solid State Ionics.

[cit21] Jung S.-Y., Rajagopal R., Ryu K.-S. (2020). J. Energy Chem..

[cit22] Ohtomo T., Hayashi A., Tatsumisago M., Kawamoto K. (2013). Electrochemistry.

[cit23] Tsukasaki H., Morimoto H., Mori S. (2020). Solid State Ionics.

[cit24] Huang B., Yao X., Huang Z., Guan Y., Jin Y., Xu X. (2015). J. Power Sources.

[cit25] Tao Y., Chen S., Liu D., Peng G., Yao X., Xu X. (2015). J. Electrochem. Soc..

[cit26] Guo Y., Guan H., Peng W., Li X., Ma Y., Song D., Zhang H., Li C., Zhang L. (2020). Solid State Ionics.

[cit27] Ohtomo T., Hayashi A., Tatsumisago M., Kawamoto K. (2013). J. Solid State Electrochem..

[cit28] Hayashi A., Muramatsu H., Ohtomo T., Hama S., Tatsumisago M. (2014). J. Alloys Compd..

[cit29] Ohtomo T., Hayashi A., Tatsumisago M., Kawamoto K. (2013). J. Non-Cryst. Solids.

[cit30] Hayashi A., Muramatsu H., Ohtomo T., Hama S., Tatsumisago M. (2013). J. Mater. Chem. A.

[cit31] Li J., Chen H., Shen Y., Hu C., Cheng Z., Lu W., Qiu Y., Chen L. (2019). Energy Storage Mater..

[cit32] Wang G., Lin C., Gao C., Dong P., Liang B., Shen X., Jiao Q. (2022). Electrochim. Acta.

[cit33] Wang G., Dong P., Liang B., Lin C., Shen X., Dai S., Jiao Q. (2022). J. Am. Ceram. Soc..

[cit34] Mirmira P., Zheng J., Ma P., Amanchukwu C. V. (2021). J. Mater. Chem. A.

[cit35] Kudu Ö. U., Famprikis T., Cretu S., Porcheron B., Salager E., Demortiere A., Courty M., Viallet V., Le Mercier T., Fleutot B. (2022). Energy Storage Mater..

[cit36] Liu G., Xie D., Wang X., Yao X., Chen S., Xiao R., Li H., Xu X. (2019). Energy Storage Mater..

[cit37] Zhao R., Hu G., Kmiec S., Gebhardt R., Whale A., Wheaton J., Martin S. W. (2021). ACS Appl. Mater. Interfaces.

[cit38] Dietrich C., Weber D. A., Sedlmaier S. J., Indris S., Culver S. P., Walter D., Janek J., Zeier W. G. (2017). J. Mater. Chem. A.

[cit39] Minami K., Mizuno F., Hayashi A., Tatsumisago M. (2008). J. Non-Cryst. Solids.

[cit40] Yamane H., Shibata M., Shimane Y., Junke T., Seino Y., Adams S., Minami K., Hayashi A., Tatsumisago M. (2007). Solid State Ionics.

[cit41] Quan Z., Hirayama M., Sato D., Zheng Y., Yano T. a., Hara K., Suzuki K., Hara M., Kanno R. (2017). J. Am. Ceram. Soc..

[cit42] Hood Z. D., Kates C., Kirkham M., Adhikari S., Liang C., Holzwarth N. A. (2016). Solid State Ionics.

[cit43] Gobet M., Greenbaum S., Sahu G., Liang C. (2014). Chem. Mater..

[cit44] Murakami M., Shimoda K., Shiotani S., Mitsui A., Ohara K., Onodera Y., Arai H., Uchimoto Y., Ogumi Z. (2015). J. Phys. Chem. C.

[cit45] Schmedt auf der Günne J., Eckert H. (1998). Chem.–Eur. J..

[cit46] Neuberger S., Culver S. P., Eckert H., Zeier W. G., auf der Günne J. S. (2018). Dalton Trans..

[cit47] Tufail M. K., Ahmad N., Zhou L., Faheem M., Yang L., Chen R., Yang W. (2021). Chem. Eng. J..

[cit48] Zhao F., Alahakoon S. H., Adair K., Zhang S., Xia W., Li W., Yu C., Feng R., Hu Y., Liang J. (2021). Adv. Mater..

[cit49] Zhou L., Tufail M. K., Yang L., Ahmad N., Chen R., Yang W. (2020). Chem. Eng. J..

[cit50] Seino Y., Nakagawa M., Senga M., Higuchi H., Takada K., Sasaki T. (2015). J. Mater. Chem. A.

[cit51] Minami K., Hayashi A., Tatsumisago M. (2011). J. Am. Ceram. Soc..

[cit52] Tsukasaki H., Morimoto H., Mori S. (2019). J. Power Sources.

[cit53] Ohtomo T., Hayashi A., Tatsumisago M., Kawamoto K. (2013). J. Solid State Electrochem..

[cit54] Vicente-Serrano S. M., Nieto R., Gimeno L., Azorin-Molina C., Drumond A., El Kenawy A., Dominguez-Castro F., Tomas-Burguera M., Peña-Gallardo M. (2018). Earth Syst. Dyn..

[cit55] Sharafi A., Yu S., Naguib M., Lee M., Ma C., Meyer H. M., Nanda J., Chi M., Siegel D. J., Sakamoto J. (2017). J. Mater. Chem. A.

[cit56] Li Y., Chen M., Liu B., Zhang Y., Liang X., Xia X. (2020). Adv. Energy Mater..

[cit57] Uvarov N., Isupov V., Sharma V., Shukla A. (1992). Solid State Ionics.

[cit58] Sakuda A., Takeuchi T., Kobayashi H. (2016). Solid State Ionics.

[cit59] Wan H., Mwizerwa J. P., Han F., Weng W., Yang J., Wang C., Yao X. (2019). Nano Energy.

