# Supplementary material for: Improved air-stability and conductivity in the 75Li2S·25P2S5 solid-state electrolyte system: the role of Li7P3S11
Source: RSC Adv. 2023 Sep 8;13(39):27066–76. doi: 10.1039/d3ra04706g (PMC10488319; doi:10.1039/d3ra04706g)
Supplement: RA-013-D3RA04706G-s001 [file RA-013-D3RA04706G-s001.pdf]

Electronic Supplementary Material (ESI) for RSC Advances.
This journal is © The Royal Society of Chemistry 2023

Supporting Information

Improved air-stability and conductivity in the $75Li_2S{\cdot}25P_2S_5$ solid-state electrolyte system: the role of $Li_7P_3S_{11}$

Chen Mi[a], Simon R. Hall[a*]

[a]School of Chemistry, Cantock's Close, University of Bristol, Bristol BS8 1TS

*email: simon.hall@bristol.ac.uk

This file includes:

Supplementary Text

Tables S1 to S14

Figs S1 to S8

Supplementary Text

The calculation of ionic conductivity and activation energy of the samples

Ionic conductivity (σ) values were calculated by Eq. (S1) using resistance values obtained from the EIS.

$$\sigma = d/RS \quad \text{(S1)}$$

where d (cm) is the thickness of solid state electrolytes, S (cm^{-2}) belongs to the effective electrolyte area, and R represents the total electrolyte resistance.

In addition, activation energies (Ea) could be calculated using Eq. (S2),

$$\sigma = \sigma_0 \exp(-Ea/RT) \quad \text{(S2)}$$

where σ_0 is the pre-exponential factor, R is the gas constant, and T is the absolute temperature.

Exposure method A for pXRD

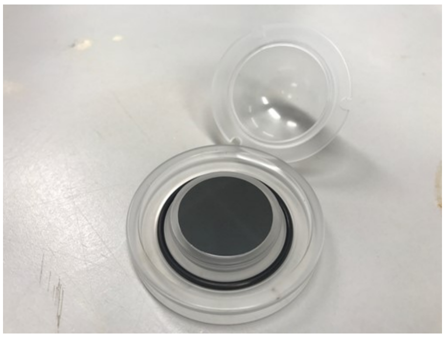

Perform pXRD experiments using the silicon bubble holder as shown in the picture to ensure that the sample is in a sealed state during the testing process. First, the sample was prepared in a glove box filled with nitrogen. This sample was called 0 min (corresponding to the mark in Fig. 10). After the test, the bubble holder lid was opened to expose the sample to the air environment, quickly placed it in the glove box chamber with a volume of approximately 30000 cm^3, and quickly tightened the chamber lid (the entire process took about 10 seconds). The glove box chamber has been opened and filled with air for 1 minute in advance. After 1 minute in the air-filled chamber, the sample was transferred to the glove box and covered. This sample was named 1 min. In the same way, the sample was placed back into the glove box chamber again after testing and left for 1 minute (being exposed to air for a total of 2 minutes). This

sample was called 2 min. Following that, the sample was exposed to air for another 1 minute, 2 minutes, 2 minutes, and 3 minutes, and was given the names 3 min, 5 min, 7 min, and 10 min.

Exposure method B for pXRD

In the glove box, 0.1g of sample was placed in each of the four vials with a diameter of 10 mm and a volume of 28 ml. Then, the vials were sealed and removed from the glove box. In the fume hood, the vial lids were opened and the samples were exposed to air for 1 day, 2 days, 3 days and 7 days, respectively.

Exposure method A for SEM

The tested sample was always placed in the SEM chamber with a volume of 38000 cm^3 until the test was completed. The exposure time of the sample in the air was controlled by vacuuming and venting the SEM chamber via the SEM console. Since vacuuming and venting are both gradual processes, timing commenced when they were finally finished. The vacuuming and venting processes took about 3 minutes in total.

Exposure method B for SEM

The sample preparation is the same as **Exposure method B for pXRD**.

Supplementary Tables

Table S1. T_g, T_c and T_c - T_g of ball-milled (100-x)(0.75Li_2S·0.25P_2S_5)·xP_2O_5 (x = 0, 1, 2, 3, 4).

Sample	T_g / °C	T_c / °C	T_c - T_g / °C
x = 0 [1]	178.4	220.1	41.7
x = 1	180.2	223.1	42.9
x = 2	181.8	225.5	43.7
x = 3	183.0	227.3	44.3
x = 4	184.5	229.7	45.2

Table S2. Peak assignments for the 0PO [1].

Raman shift / cm^{-1}	Assignment	Ref
269	δ_{def}(S-P-S) in PS_4^{3-}	[2]
388	$P_2S_6^{4-}$	[3]
423	$v_s(PS_4^{3-})$ in PS_4^{3-}	[4]
574	$v_{as}(PS_4^{3-})$ in PS_4^{3-}	[4]

Table S3. Peak assignments for the 1PO.

Raman shift / cm^{-1}	Assignment	Ref
266	δ_{def}(S-P-S) in PS_4^{3-}	[2]
387	$P_2S_6^{4-}$	[3]
422	PS_4^{3-} and $P_2S_7^{4-}$	[4-5]
573	$v_{as}(PS_4^{3-})$ in PS_4^{3-}	[4]

Table S4. Peak assignments for the 2PO.

Raman shift / cm^{-1}	Assignment	Ref
266	δ_{def}(S-P-S) in PS_4^{3-}	[2]
387	$P_2S_6^{4-}$	[3]
417	PS_4^{3-} and $P_2S_7^{4-}$	[4-5]
573	$v_{as}(PS_4^{3-})$ in PS_4^{3-}	[4]

Table S5. Peak assignments for the 3PO.

Raman shift / cm^{-1}	Assignment	Ref
266	δ_{def}(S-P-S) in PS_4^{3-}	[2]
385	$P_2S_6^{4-}$	[3]
419	PS_4^{3-} and $P_2S_7^{4-}$	[4-5]
573	$v_{as}(PS_4^{3-})$ in PS_4^{3-}	[4]

Table S6. Peak assignments for the 4PO.

Raman shift / cm^{-1}	Assignment	Ref
221	S-S-S bending in S_8	[2]
266	δ_{def}(S-P-S) in PS_4^{3-}	[2]
387	$P_2S_6^{4-}$	[3]
419	PS_4^{3-} and $P_2S_7^{4-}$	[4-5]
471	S-S bending in S_8	[3]
573	$v_{as}(PS_4^{3-})$ in PS_4^{3-}	[4]

Table S7. The fitting parameters of deconvoluted Raman spectra of 1PO. FWHM is an abbreviation for full width of the peak at half maximum value.

Assigned unit	Peak center / cm^{-1}	FWHM / cm^{-1}	Area / a.u.
$P_2S_6^{4-}$	387	8.6	743235
$P_2S_7^{4-}$	419	6.7	43343
PS_4^{3-}	423	5.3	191323

Table S8. The fitting parameters of deconvoluted Raman spectra of 2PO.

Assigned unit	Peak center / cm^{-1}	FWHM / cm^{-1}	Area / a.u.
$P_2S_6^{4-}$	387	8.0	609276
$P_2S_7^{4-}$	416	4.6	49615
PS_4^{3-}	418	5.1	110409

Table S9. The fitting parameters of deconvoluted ^{31}P MAS NMR of 0PO. (G)PS_4^{3-}, (C)PS_4^{3-}, $P_2S_6^{4-}$ are the abbreviations for PS_4^{3-} (glass), PS_4^{3-} (crystal) and $P_2S_6^{4-}$ (glass + crystal), respectively.

Assigned unit	Peak center / ppm	FWHM / ppm	Area / a.u.
(G)PS_4^{3-}	87.9	7.1	1.4
(C)PS_4^{3-}	86.6	0.8	1.2
$P_2S_6^{4-}$	106.0	7.7	5.1

Table S10. The fitting parameters of deconvoluted ^{31}P MAS NMR of 1PO. $P_2S_7^{4-}$ is the abbreviations for $P_2S_7^{4-}$ (crystal).

Assigned unit	Peak center / ppm	FWHM / ppm	Area / a.u.
(G)PS_4^{3-}	86.6	15.5	1.3
(C)PS_4^{3-}	86.7	0.8	1.2
$P_2S_7^{4-}$	88.8	4.2	1.6
$P_2S_6^{4-}$	105.9	7.7	2.4

Table S11. The fitting parameters of deconvoluted ^{31}P MAS NMR of 2PO.

Assigned unit	Peak center / ppm	FWHM / ppm	Area / a.u.
(G)PS_4^{3-}	86.7	7.8	3.0
(C)PS_4^{3-}	86.7	0.6	0.7
$P_2S_7^{4-}$	90.8	5.8	2.5
$P_2S_6^{4-}$	105.6	6.5	0.7

Table S12. The fitting parameters of deconvoluted ^{31}P MAS NMR of 3PO.

Assigned unit	Peak center / ppm	FWHM / ppm	Area / a.u.
(G)PS_4^{3-}	86.6	13.7	0.8
(C)PS_4^{3-}	86.6	1.0	1.6
$P_2S_7^{4-}$	89.6	2.7	0.3
$P_2S_6^{4-}$	106.1	7.9	1.9

Table S13. The fitting parameters of deconvoluted ^{31}P MAS NMR of 4PO.

Assigned unit	Peak center / ppm	FWHM / ppm	Area / a.u.
(C)PS_4^{3-}	86.6	1.1	1.7
$P_2S_7^{4-}$	89.7	2.8	0.4
$P_2S_6^{4-}$	105.9	7.4	4.2

Table S14. The ionic conductivity of 2PO at various temperatures.

Temperature / °C	Thickness / cm	Area / cm^2	Resistance / Ω	Ionic conductivity / S cm^{-1}
25	0.1	0.785	2032	6.3×10^{-5}
70	0.1	0.785	735	1.7×10^{-4}
80	0.1	0.785	612	2.1×10^{-4}
90	0.1	0.785	509	2.5×10^{-4}
100	0.1	0.785	406	3.1×10^{-4}
110	0.1	0.785	331	3.8×10^{-4}
120	0.1	0.785	280	4.5×10^{-4}

Supplementary Figures

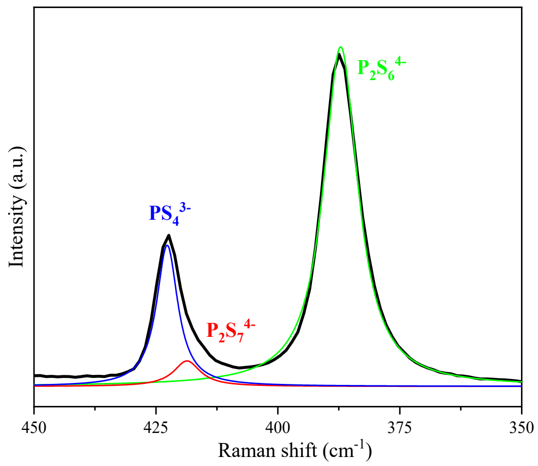

Fig. S1. Deconvoluted Raman spectra of 1PO. The black line represents the experimental data. The blue, green and red lines are the deconvoluted signals attributed to PS_4^{3-}, $P_2S_6^{4-}$ and $P_2S_7^{4-}$ moieties, respectively.

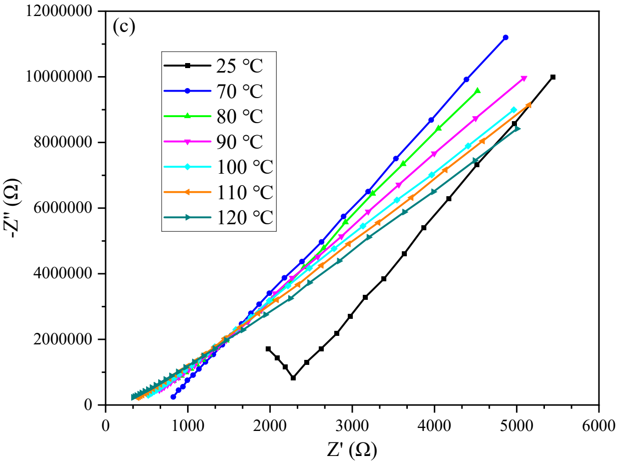

Fig. S2. The Nyquist plot of electrochemical impedance for the 2PO at various temperatures.

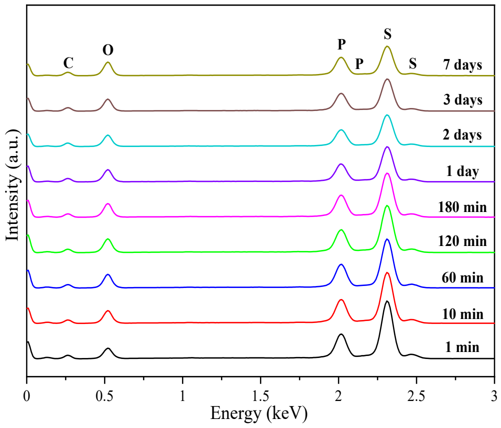

Fig. S3. EDS spectra of 2PO exposed at various times.

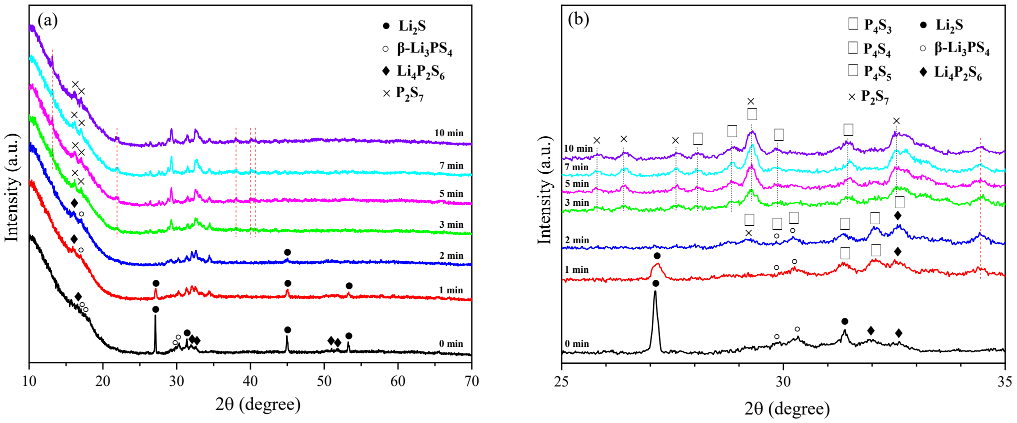

Fig. S4. (a) pXRD patterns of 0PO exposed to air for 1 min, 2 min, 3 min, 5 min, 7 min and 10 min. (b) is an enlarged view of (a) in the 2θ of 25° to 35° range.

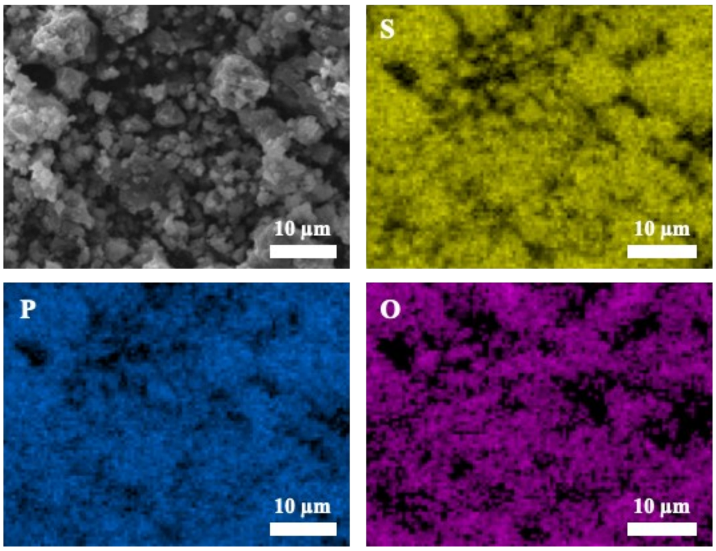

Fig. S5. EDS elemental mapping of 2PO within a chosen area.

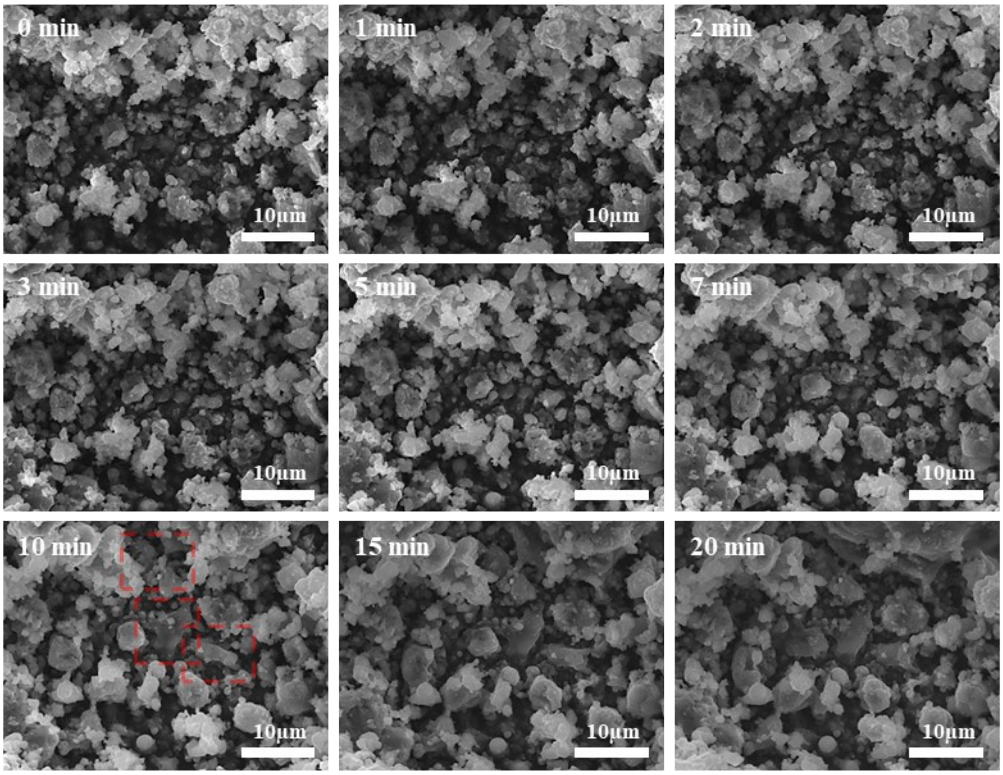

Fig. S6. Typical SEM images of 2PO at various exposure times using exposure method A.

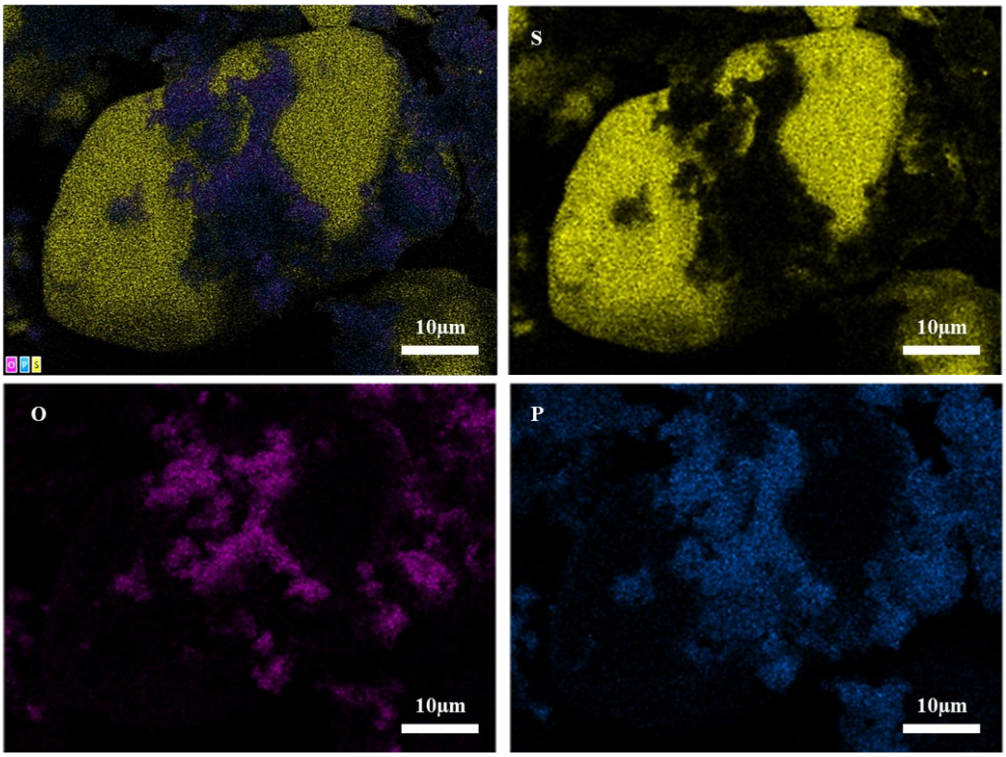

Fig. S7. EDS elemental mapping of 2PO exposed to air for 2 days.

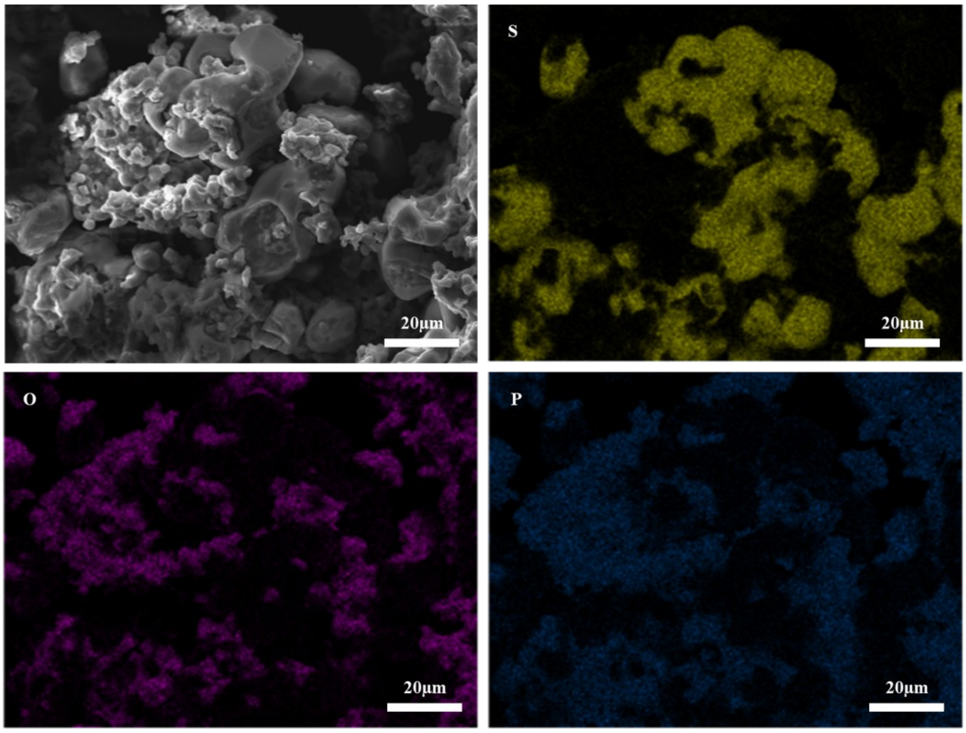

Fig. S8. SEM and EDS images of 2PO exposed to air for 7 days.

References

[1] C. Mi and S. R. Hall, *Solid State Ionics* **2023**, *389*, 116106.

[2] Y. Zhou, C. Doerrer, J. Kasemchainan, P. G. Bruce, M. Pasta and L. J. Hardwick, *Batteries & Supercaps* **2020**, *3*, 647-652.

[3] Z. Quan, M. Hirayama, D. Sato, Y. Zheng, T. a. Yano, K. Hara, K. Suzuki, M. Hara and R. Kanno, *Journal of the American Ceramic Society* **2017**, *100*, 746-753.

[4] L. Sang, R. T. Haasch, A. A. Gewirth and R. G. Nuzzo, *Chemistry of Materials* **2017**, *29*, 3029-3037.

[5] Ö. U. Kudu, T. Famprikis, S. Cretu, B. Porcheron, E. Salager, A. Demortiere, M. Courty, V. Viallet, T. Le Mercier and B. Fleutot, *Energy Storage Materials* **2022**, *44*, 168-179.
